# Evaluation of Thermal Properties of Composites Prepared from Pistachio Shell Particles Treated Chemically and Polypropylene

**DOI:** 10.3390/molecules27020426

**Published:** 2022-01-10

**Authors:** Beatriz Adriana Salazar-Cruz, María Yolanda Chávez-Cinco, Ana Beatriz Morales-Cepeda, Claudia Esmeralda Ramos-Galván, José Luis Rivera-Armenta

**Affiliations:** Tecnológico Nacional de México/Instituto Tecnológico de Ciudad Madero, Centro de Investigación en Petroquímica, 89600 Altamira, Tamaulipas, Mexico; beatriz.sc@cdmadero.tecnm.mx (B.A.S.-C.); maria.cc@cdmadero.tecnm.mx (M.Y.C.-C.); abmorales@itcm.edu.mx (A.B.M.-C.); claudia.rg@cdmadero.tecnm.mx (C.E.R.-G.)

**Keywords:** chemical treatment, crystallinity, pistachio shell particles, thermal stability

## Abstract

The purpose of the present work was to prepare polypropylene (PP) matrix composited filled with chemically treated pistachio shell particles (PTx), and evaluate their effect on the composites’ thermal properties. PP-PTx composites were formulated in different PTx content (from 2 to 10 phr) in a mixing chamber, using the melt-mixing process. The PTx were chemically treated using a NaOH solution and infrared spectroscopy (FTIR). According to thermogravimetric analysis (TGA), the treatment of pistachio shell particles resulted in the remotion of lignin and hemicellulose. The thermal stability was evaluated by means of TGA, where the presence of PTx in composites showed a positive effect compared with PP pristine. Thermal properties such as crystallization temperature (Tc), crystallization enthalpy (∆Hc), melting temperature (Tm) and crystallinity were determinate by means differential scanning calorimetry (DSC); these results suggest that the PTx had a nucleation effect on the PP matrix, increasing their crystallinity. Dynamic mechanical analysis (DMA) showed that stiffness of the composites increase compared with that PP pristine, as well as the storage modulus, and the best results were found at a PTx concentration of 4 phr. At higher concentrations, the positive effect decreased; however, they were better than the reference PP.

## 1. Introduction

Using polymer materials that perform better when combined with natural resources has attracted interest among research groups preparing composite materials. Agro-industrial waste is an option for developing new low-cost materials derived from renewable sources [1]. The use of natural additives obtained from agro-industrial waste in polymer matrices has gained interest in recent years because they take advantage of materials that do not have an industrial application and reduce environmental pollution while improving specific mechanical and thermal properties [2,3]. One of the main applications of agro-industrial waste materials is as absorbents of polluting metals such as chromium; their absorption capacity of waste materials has been improved by treating them with acidic aqueous solutions [4]. Furthermore, the absorption properties of walnut shells have also been studied [5], finding an improved sorption capacity due to the presence of functional groups in the shells after the modification process. Another application of this kind of material is as a source of cellulose nanocrystals which are highly appreciated [6].

Seed husks are one of the most promising agro-industrial waste materials for research since they are used as reinforcements in polymer matrices. For instance, pistachio has been used as an additive in polymer matrices such as high density polyethylene (HDPE) [7], polymehtly methacrylate (PMMA) [8,9], styrene butadiene rubber (SBR) [10]; polyester resins [11], epoxy resins [12], and polypropylene (PP) [13,14], these studies have reported improvements in the polymers’ mechanical, thermal, and aging properties. Other types of seeds such as almond shells [15]; Jatropha curcas [16]; pumpkin seed shells [17], cashew nut [18,19], and mango seeds [20] have been studied, reporting significant improvements in the mechanical properties of polymer matrices. The additives have acted as nucleation agents favoring the crystallization process. However, few reports exist in which agro-industrial waste materials have been chemically treated before adding them to the polymer matrices.

The chemical treatment of materials of natural origin has been deeply studied to modify their natural hydrophilic character since they are materials of lignocellulosic origin and contain strongly polarized hydroxyl groups. One of the treatments applicable to natural materials is an alkaline treatment with NaOH solutions at various concentrations. This treatment is prevalent in cellulosic fibers, and it has been recently used in seed particles [18,19]. In these studies, an alignment effect of the fibers treated with NaOH has been found, which is reflected in improving their dynamic mechanical properties, such as increasing their storage modulus and decreasing their tan δ. Cane baggasse has also been treated with NaOH solutions, generating changes in the thermal stability of polymeric composites [21,22]. Another type of shell studied and modified through a chemical treatment is the cocoa shell [23]. These shells were treated with diazonium salts grafted in the seeds producing a surface modification that was reflected in improving their mechanical properties [24]; this behavior makes them highly compatible with polymers.

Aspirila et al. [25] studied the addition of Jatropha curcas particles to a vinyl ester polymer, enhancing the thermal stability of a composite as a result of the Jatropha particles incorporation. The effect of acidic hydrolysis on the physical and chemical properties of Jatropha curcas has also been studied previously, showing that sulfuric acid can successfully modify the properties of polymers [26]. Other studies have reported a variety of chemical treatments for modifying natural materials to reinforce polymeric composites. For example, surface modification with alkaline treatments using silanated agents acting as coupling agents, acetyl groups reacting with cellulose hydroxy groups, benzoylation with benzoyl chloride for reducing hydrophilicity and improving interfacial adhesion, peroxide treatments, and grafting reactions, are some of the most commonly reported treatment processes [27].

Polypropylene (PP) is a polymer with a wide variety of applications, for instance, the fabrication of furnishing, domestic pieces, raffia fibers, as well as in the automotive industry; however, when it is exposed to environmental oxygen, it undergoes partial degradation. Some natural waste materials such as fruit fibers and shells have been employed as PP additives, reporting improvements in the polymer properties, such as flame-retarding [28].

Therefore, the effect of an alkaline treatment of pistachio shell particles is investigated in this study. The pistachio particles were added at different concentrations, ranging from 2 to 10 phr, into the composite formulation using PP as a polymeric matrix. The thermal properties of the composites were evaluated by differential scanning calorimetry (DSC), thermogravimetric analysis (TGA) and dynamic mechanical analysis (DMA).

## 2. Results

Following the obtained results from thermal analytic techniques are presented.

### 2.1. Thermogravimetric Analysis (TGA) for Pistachio Shell Particles

**Figure 1:**
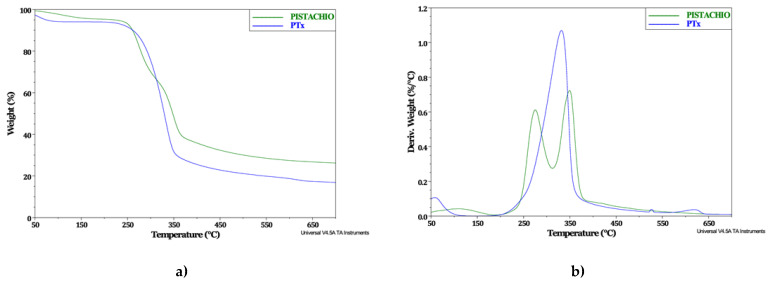
TGA thermogram of pistachio shell particles without and treated chemically (PTx) (**a**), and derivative thermogravimetric (DTG) curve (**b**).

The thermal stability of the pistachio shell particles was evaluated by means of TGA. Figure 1a shows the thermograms comparing the thermal behavior of the pistachio particles treated with NaOH 1 M (PTx), with the untreated pistachio particles, in order to detect any changes induced by the chemical treatment process over the thermal properties. The untreated pistachio particles exhibit two decomposition stages and a greater residue than the PTx, which shows a single weight loss. This behavior can be associated with the chemical treatment removing some components such as lignin and hemicellulose, which have a wide range for decomposition (from 190 to 900 °C) due to its complex and heterogeneous structure [29]. Previous works report that degradation of pistachio shell is divided into three stages: drying, devolatilization and charring [30]. The proportions of hemicellulose, cellulose and lignin are hard to be identified by means of derivative thermogravimetric (DTG) curves due to the components decomposition is carried out at similar temperatures, especially the lignin [31].

A DTG curve (Figure 1b) was generated for identifying the samples’ decomposition temperatures. The temperature of the maximum mass loss can be determinate from the maximum of the peak. The untreated pistachio particles presented two main decomposition temperatures around 275 °C and 350 °C, associated with hemicellulose degradation, as well as that of lignin and cellulose, respectively [13,15,21]. On the other hand, the PTx single decomposition step occurs at 330 °C, approximately, which indicates that the hemicellulose was removed from the particles after the chemical, leaving behind just cellulose, which decomposition takes place at a temperature of 330 °C according to the literature [32]. The material’s decomposition speed can be inferred from the intensity of its peaks; in this case, the PTx shows a higher peak compared with untreated pistachio shell particles indicating that PTx are more susceptible to thermal degradation.

**Figure 2:**
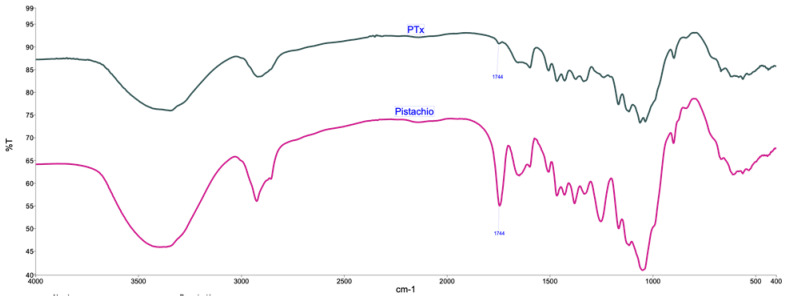
FTIR spectra for Pistachio and Pistachio treated (PTx).

Figure 2 shows the Fourier transform infrared (FTIR) spectra of the pistachio and PTx, where the characteristic peaks of each are observed, the pistachio has the broad peak around 3350 cm^−1^, attributed to OH groups, 1050 and 1412 cm^−1^ due to C-O stretching of anhydroglucose ring groups, around 2920 and 2820 cm^−1^ of C-H of symmetric and antisymmetric vibrations of groups of methyl and methylene vibrations, and peak around 1700 and 1263 cm^−1^ attributed to C-O stretching for carboxyl group of lignin and hemicellulose [12,13]. On the other hand, the PTx shows a decreasing of peaks around 1700 and 1260 cm^−1^ which are mainly associated with lignin, is not present in PTx confirming the observation in TGA, that treatment with NaOH solution causes a remotion of hemicellulose of pistachio shell particles.

### 2.2. Thermogravimetric Analysis (TGA) for PP-PTx Composites

**Figure 3:**
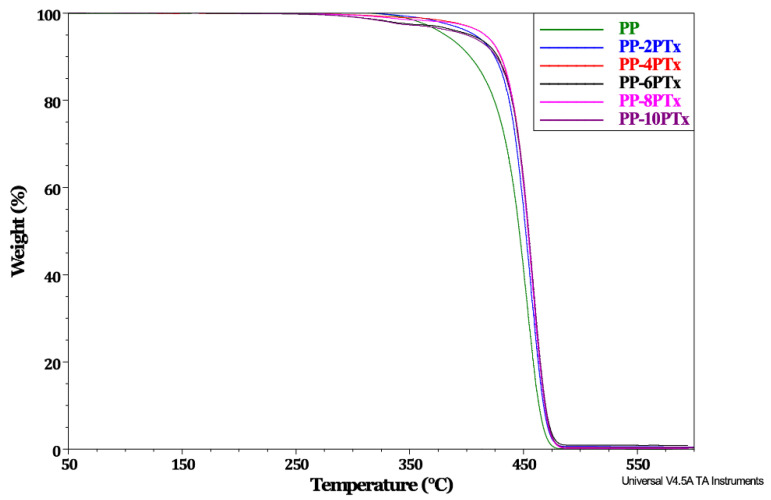
TGA thermogram of PP and PP-PTx composites.

Figure 3 shows the thermograms of PP and PP-PTx composites. As observed in Figure 3, the decomposition onset of PP arises around 374 °C, and it ends up to 500 °C, which is similar behavior to that described in reference studies [33]. Conversely, the decomposition onset of the composites occurs at 408 °C, 405 °C, 411 °C, 416 °C, and 410 °C, for the PP-2PTx, PP-4PTx, PP-6PTx, PP-8PTx, and PP-10PTx, which are higher than that of the pure PP. Karagaac [10] reported that the thermal stability of composites prepared from pistachio particles was negatively affected when concentrations above 10 phr were used; however, in that study, the particles were not subjected to any chemical treatment, and therefore, the different thermal stability observed in our study may be attributed to the treatment of the pistachio particles. On the other hand, the residue at the end of the analysis does not change as the PTx content increases. There are studies that report an increase in residue when seed husk particles are used as fillers [34]. Aprilia et al. [25], found that this increase in residue is associated with the incorporation of a high filler content, which is reflected in improved thermal stability.

Najafabadi et al. [7] recommended adding up to 40%wt. of pistachio particles to a composite, but they only reported their effect on the mechanical properties of the composite without any thermal evaluation. A thermal stability improvement on composites was reported when bamboo fibers were used as a filler [33]. However, in contrast, other studies have found that the agroindustrial waste particles added to a PP matrix [34] do not enhance the thermal stability of the composites.

**Figure 4:**
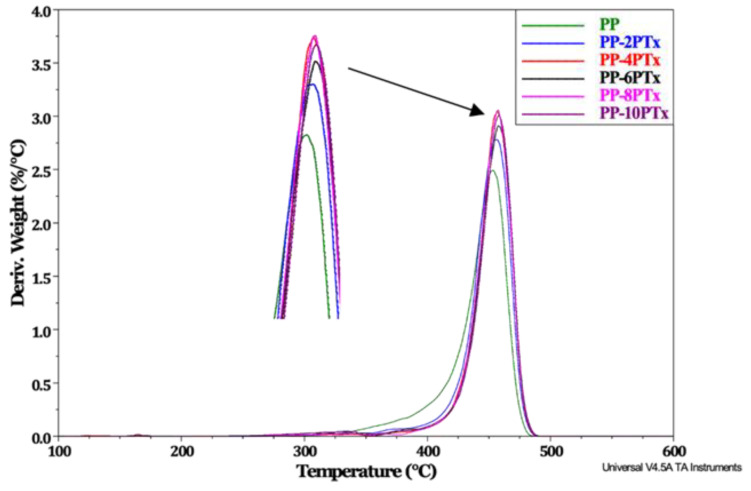
DTG thermogram of PP and PP-PTx composites.

To better observe the effect of the addition of the treated particles in the composite, the DTG curve with respect to temperature is presented in Figure 4, which shows that the PP has an average decomposition temperature around 450 °C, a similar value was previously reported [35]. When PTx particles are added to polymer there is no significant change in average decomposition temperature, but there is a change in peak height, increasing as the particle content of PTx augmented, making composites more susceptible to decomposition, e.g., the speed, also increased as the content of PTx raised. This behavior is observed because the PTx components (mainly cellulose) decompose early than PP. However, it is interesting that the PTx decomposition step was not observed in composites, which suggests that there is a good interaction with the PP matrix, due other works report the decomposition of lignocellulosic materials between 250 and 390 °C [13,21,35].

A different behavior was reported in previous studies, finding that the presence of char cashew nut shell added to the PP matrix forms a protective layer, which acts as an insulator retarding the thermal degradation of composites [36]. Kabir et al. [27] reports that thermal stability of polymer composites can be improved by remotion of hemicellulose and lignin present in agroindustrial wastes by chemical treatment.

### 2.3. Differential Scanning Calorimetry (DSC) of Composites

**Figure 5:**
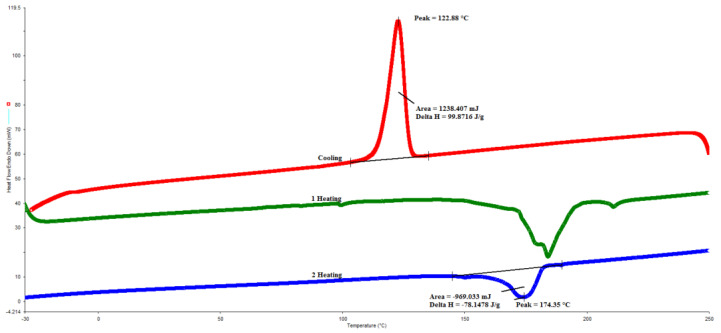
DSC thermogram of PP in a heating–cooling–heating cycle.

On the other hand, the effect of the addition of PTx on the crystallization and fusion of the composites was evaluated by DSC. Figure 5 shows the DSC thermogram of PP, and Table 1 reports the values of crystallization temperature (Tc), crystallization enthalpy (∆H_c_), melting temperature (T_m_), and fusion enthalpy (∆H_m_). The PP-PTx composites DSC thermograms show the same behavior as only PP. It is observed that the pure PP has a crystallization temperature of 123 °C, with a ∆H_c_ = 99.87 J/g, while the melting temperature is 174 °C with a ∆H_m_ = 78.14 J/g. These results prove that the more PTx content, the greater the composites melting temperature and ∆Hm, indicating that a larger number of crystalline zones were present, with the PP-6 PTx composite exhibiting the highest values. Therefore, the PTx acted as a nucleating agent ordering the polymer chains, which was reflected as an increase of up to 6 °C in the melting temperature. This behavior was not previously reported when seed particles were used as fillers in PP matrices, so this finding is relevant. This behavior is attributed to a better interfacial interaction between cellulose that acts as a nucleating agent for PP, generating a transcrystalline region around PTx. Quillin et al. [37] reported similar behavior when studying the nucleating effect of chemically treated cellulose fibers in a PP matrix.

The melting temperature of a crystallizable polymer is critical in analyzing nucleation kinetics; however, this is a complicated process. The difficulty lies in the limited sizes of crystallites that are smaller than the length of the chains. The process of crystallization of PP depends a lot on the crystallization conditions and the molecular weight of the polymer [38]. According to the results obtained, the crystallinity process in PP-PTx composites is not affected, as the Tc did not change being almost the same 123 °C, but the ∆Hc show a significant change being lower when PTx increase in the composite.

Furthermore, the crystallization temperature is similar to that reported in a previous PP study [34], where PP-wood flour composites with a particle size of approximately 147 microns were studied. Similar values to those presented in our work were obtained in the reference study; they also found that wood particles induce the crystallization of the PP matrix and that the shear stress promotes the beta phase. In our study, the crystallization process was not affected with the addition of PTx, since the crystallization temperature showed a constant behavior, and the crystallization enthalpy remained very similar up to a concentration of 6 phr; after that point, an increase in the concentration of PTx resulted in a decrease of the crystallization enthalpy. Nayak et al. [33] reported that the crystallization process in composites reinforced with bamboo fibers generates heterogeneous nucleation, thus improving the compatibility between the PP matrix and the fibers, the crystallization temperature increased due to the presence of bamboo fibers using a compatible agent, which is described by a heterogeneous nucleation effect.

Rojas-Lema et al. [12] reported that the addition of pistachio particles using a compatibility agent increases the crystallinity of polybutylene succinate in addition to favorably modifying the viscoelastic properties of the composite.

The presence of lignocellulosic particles used as fillers favors the crystallization of a polymer, although there is no significant change in its Tm. However, in these studies, the contents of shell particles were up to 40% [13]. This behavior indicates that particles of lignocellulosic fillers generate a nucleate effect, just as calcium carbonate and distiller grains work, which would make the use of PTx interesting in the industrial field. Another way in which this nucleating effect can be favored is by adding a compatibility agent that improves the interactions between the particles and the polymer matrix [39]. This effect of nucleating effect was reported for polyvinylpyrrolidone and sodium liquid glass, increasing the degree of crystallinity on a PP matrix [40].

### 2.4. Dynamic Mechanical Analysis (DMA) of Composites

**Figure 6:**
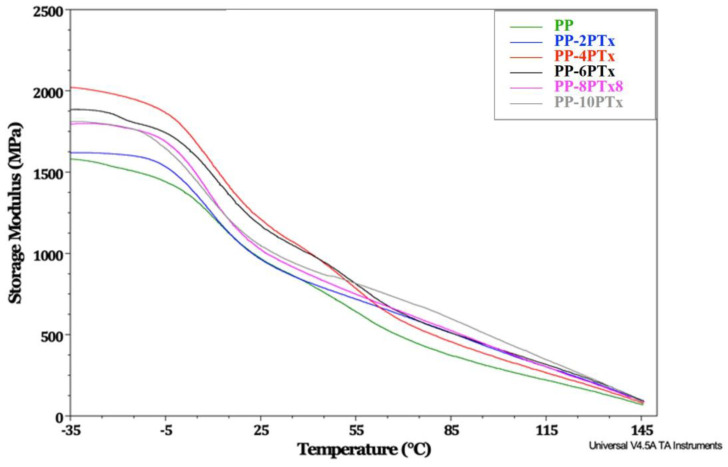
DMA thermogram of Storage Modulus for PP and PP-PTx composites.

Figure 6 shows the DMA thermogram of PP and PP-PTx composites. It is observed that the PP storage modulus is lower at the beginning of the analysis, compared to those of the PP-PTx composites, with the highest value corresponding to the PP-4PTx sample. This result implies that the material is able to withstand energy, such as loads. Even though the storage module decreases at concentrations greater than 4 phr, it does not reach the value of pure PP; therefore, the addition of PTx generates a positive reinforcement effect on the PP matrix, especially at low concentrations. The increase in the storage modulus is related to the rigidity of a material due to the presence of a rigid filler in a semi-rigid matrix, and the particles introduce a high degree of mechanical restriction, which is reflected in a reduction in the mobility and deformability of the matrix; on the other hand, the decrease in the storage modulus, compared to that of the pristine PP in the transition region, can be an indicator of agglomeration problems of the particles due to high concentrations [34]. An important observation is that at temperatures around 60 °C, as the content of PTx increases, the storage modulus of the composite is higher, which implies that the effect of the modification of pistachio particles becomes more evident at temperatures above 60 °C, which is interesting for defining the final application of the composites; however, in the entire studied range of temperatures, the storage module is always higher for all the composites compared to that of pristine PP.

Salasinska and Ryszkowska [8] also reported an increase in the value of the storage modulus by adding pistachio husks and sunflower husk grains to a polymeric matrix, attributing this behavior to the good adhesion between the particles and the polymer; however, in these studies, the particles were not subjected to any chemical treatment. On the other hand, there are reports of almond shell particles that, depending on the type of treatment they undergo, may present an increase in the storage module when they are added to a PP matrix, the reported treatments were with NaOH for periods of time of up to 48 h or an etherification treatment [15]. The remaining cellulose after the NaOH treatment produces a stronger and more rigid material; this condition has been related to the variation of the storage modulus [41]. The chemical treatment to lignocellulosic fiber used as reinforcer in a polymer matrix has shown an increase in storage modulus when added to the matrix compared to when untreated fibers are used [18]. The former behavior is attributed to more significant interactions and increasing, adhesion between the polymer matrix and the fibers.

**Figure 7:**
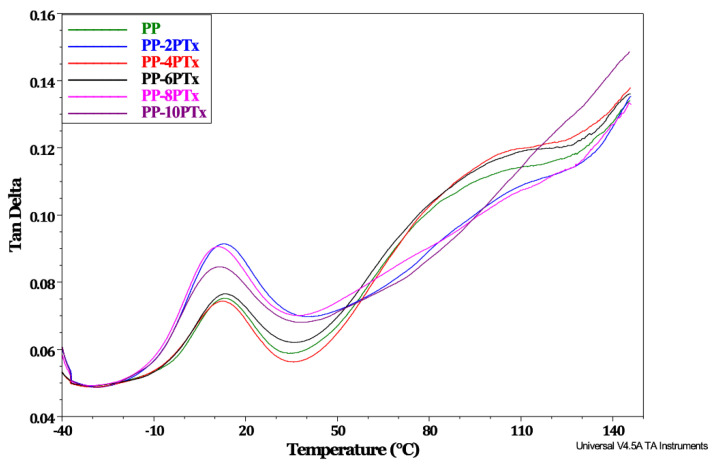
DMA thermogram of Tan δ for PP and PP-PTx composites.

The tan δ is called the damping factor and can be used to identify relaxations on the polymer matrix. The magnitude of the tan δ peak can be related to the behavior of the material in impact behavior [19]. Figure 7 shows the DMA thermogram of the tan δ curve of PP and PP-PTx composites, where the damping ratio of the composites shows to be high at elevated particle concentrations and the width of the peaks is greater compared to that of the pristine PP. The former condition is characteristic of materials having low dissipation energy, more complex structures, and reduced hardness, which can be confirmed by the fact that naturally occurring polymers with amorphous regions can be replaced with rigid lignocellulosic fillers [13]. The tan δ shows two signs of relaxation; the first is close to 10 °C, and the other is approximately at 100 °C, where the first signal is related to the Tg of the PP amorphous phase. In contrast, the second one is attributed to a laminar and rotational slipping mechanism within the crystalline phase. The first signal related to the Tg of the composites does not present a significant variation as the content of PTx particles increases. This is the opposite behavior to the one that was previously reported [34], which described that increasing the filler content, such as wood flour and talc, caused a reduction in the temperature of the peak associated with the Tg, indicating a nucleating effect between the particles which leads to a faster crystallization of the PP. Other reports have found a different effect, reporting that the maximum of tan δ has higher temperatures due to the fact that the presence of particles of natural origin inhibits the movement of the chains, which is reflected in an increase of Tg [8,40,41]. On the other hand, the height of the tan δ peak decreases as the flour content increases because the PP fraction is reduced as the presence of the particles restricts the mobility of the segments of the PP amorphous phase [42].

Moreover, the width of the tan δ peak is greater for the composites with PTx, which is associated with a more complex structure. Aziz & Ansell [17] reported an increase in the Tg of a composite modified with shell particles, attributed to the filling effect of the shell particles occupying the free spaces between the polymer chains generated; therefore, the movement of those changes is restricted and only occurs at a higher temperature.

## 3. Materials and Methods

### 3.1. Materials

The PP with a melt flow index (MFI) of 3.3 g/10 min was provided by Indelpro, Altamira, México. The pistachio shells were collected from domestic sources. The shells were washed, dried and then milled in an IKA analytical mill model A11 (Wilmington, NC, USA), to decrease their particle size to 177 µm using an ASTM sieve #80. The particles were treated with a NaOH 1M solution during 1 h under magnetic stirring at room temperature. After that time the particles were washed until neutralized, and dried under vacuum, the treated particles were identified as PTx.

### 3.2. Particles Characterization

After the treatment, PTx and pistachio shell particles were analyzed by thermogravimetric analysis (TGA) using a TA Instruments Simultaneous thermal analyzer (SDT) model Q600 (New Castle, DE, USA), in a temperature range from room temperature to 700 °C with a heating rate of 10 °C/min, in a N2 atmosphere with 100 mL/min flow. This analysis was carried out to evaluate changes in thermal behavior of particles. Fourier transform Infrared spectroscopy (FTIR) was carried out in the aim to identify the characteristic groups of cellulose and hemicellulose and corroborate the elimination of the last one in PTx. The analysis was carried out using a Perkin Elmer Spectrophotometer model Spectrum One (Waltham, MA, USA) using the KBr plates technique, with 200 mg of KBr FTIR grade, from Sigma-Aldrich, and 2 mg of samples, with a range from 4000 to 400 cm^−1^, 12 scans and a resolution of 4 cm^−1^.

### 3.3. Composites Preparation

The composites were prepared at different PTx content, and samples were identified with the following codes: PP-2PTx, PP-4 PTx, PP-6PTx, PP-8PTx and PP-10PTx, which correspond to 2, 4, 6, 8 and 10 phr of PTx content respectively. A Brabender Plasti-corder chamber (Duisburg, Germany) was used for preparing the composites for 15 min at 180 °C and 60 rpm.

### 3.4. Composites Characterization

The composites were characterized to evaluate their thermal properties. First, the thermal stability was investigated using a TA Instruments SDT analyzer model Q 600 (New Castle, DE, USA), with a heating rate of 10 °C/min, under a N2 atmosphere flowing 100 mL/min. A sample of 10 mg and a platinum crucible were used. The crystallization and melting behavior of composites were studied using a Perkin Elmer DSC, DSC8000 model (Waltham, MA, USA). The analysis was performed under a nitrogen atmosphere, with an initial heating process from −30 to 250 °C at a heating rate of 20 °C/min, the temperature was kept constant for 5 min to eliminate previous thermal history. Then, the temperature was reduced to −30 °C at a heating rate of 20 °C/min, and this temperature was kept constant for 5 min. Finally, a second heating step was carried out to increase temperature to 250 °C with a heating rate of 10 °C/min, and the degree of crystallinity (Xc) was determined by the following equation:

Xc = (∆Hf/∆H°f) × 100(1)

∆Hf is the enthalpy of fusion per unit mass of the PP, calculated from the area under the melting peak of the composite, ∆H°f is the enthalpy per unit mass of the 100% crystalline PP, with a value of 207 J/g [28].

The dynamic mechanical properties of the composites were determinate using a TA Instruments DMA model Q800 (New Castle, DE, USA), the samples’ size was 20 × 10 × 3 mm^3^, with a dual cantilever clamp, in the temperature range from −30 to 150 °C, with a heating rate of 5 °C/min and 1 Hz frequency. The storage modulus (E’) and tan δ curves were analyzed.

## 4. Conclusions

The following conclusions are based on the findings of this work: The chemical treatment of pistachio shell particles with NaOH 1M solution removes the lignin and hemicellulose from shells, according to TGA and FTIR results. The presence of PTx in PP composites increases their crystallinity and Tm, which is attributed to a nucleation effect of treated particles. The best values correspond to the sample containing 6 phr, at higher concentrations, the properties are lower, but they are still higher than those of the PP pristine. The crystallization process was not affected since the crystallization temperature did not exhibit any significant change, however, the crystallization enthalpy decrease when PTx content increased. The thermal stability of PP-PTx composites increased with the PTx content. The reported onset temperature was higher than that of pristine PP, the 8 phr composite presented the highest temperature of the experiment. On the other hand, the susceptibility to thermal degradation increased with the PTx concentration. From DMA results, the presence of PTx in the PP matrix showed a positive effect on thermomechanical behavior, increasing the stiffness and exhibiting a low energy dissipation associated with the tan δ peak. In general, the PTx proved to be an effective filler for PP since its incorporation improved the composites’ thermal properties. The overall results suggest that treated pistachio shell particles are an interesting natural additive for this kind of polymer.

## Figures and Tables

**Table 1:** Data obtained from DSC analysis for PP-PTx composites.

Sample	Tc, °C	∆Hc, J/g	Tm, °C	∆Hm, J/g	%C
PP	123	99.87	174	78.14	37.74
PP-2PTx	124	98.45	177	83.49	40.33
PP-4-PTx	123	100.03	180	81.66	39.45
PP-6PTx	123	94.26	181	85.6	41.35
PP-8PTx	123	94.89	180	77.82	37.58
PP-10PTx	124	78.45	179	64.76	31.28

## Data Availability

Not applicable.

## Sample Availability

Samples of the compounds are available from the authors.

## References

[1] Ortega F., Versino F., López O.V., García M.A. (2021). Biobased composites from agroindustrial wastes and by-products. Emergent Mater..

[2] Wahab R.A., Gowon J.A., Elias N. (2019). On the Renewable Polymers from Agro-industrial Biomass: A Mini Review. J. Indones. Chem. Soc..

[3] Maraveas C. (2020). Production of sustainable and biodegradable polymers from agricultural waste. Polymers.

[4] Komnitsas K., Zaharaki D., Pyliotis I., Vamvuka D., Bartzas G. (2015). Assessment of pistachio shell biochar quality and its potential for adsorption of heavy metals. Waste Biomass Valorization.

[5] Altun T., Pehlivan E. (2012). Removal of Cr (VI) from aqueous solutions by modified walnut shells. Food Chem..

[6] Zheng D., Zhang Y., Guo Y., Yue J. (2019). Isolation and characterization of nanocellulose with a novel shape from walnut (*Juglans regia* L.) shell agricultural waste. Polymers.

[7] Najafabadi M.A., Khorasani S.N., Esfahani J.M. (2017). High density polyethylene/pistachio shell flour/nanoclay composites-effect of accelerated weathering conditions on mechanical properties, relative brightness and total colour change. Polym. Polym. Compos..

[8] Salasinska K., Ryszkowska J. (2015). The effect of filler chemical constitution and morphological properties on the mechanical properties of natural fiber composites. Compos. Interfaces.

[9] Kadhim N.N., Hamad Q.A., Oleiwi J.K. (2020). Tensile and morphological properties of PMMA composite reinforced by Pistachio Shell powder used in denture applications. AIP Conference Proceedings.

[10] Karaağaç B. (2014). Use of ground pistachio shell as alternative filler in natural rubber/styrene–butadiene rubber-based rubber compounds. Polym. Compos..

[11] Alsaadi M., Erkliğ A., Albu-khaleefah K. (2018). Effect of Pistachio Shell Particle Content on the Mechanical Properties of Polymer Composite. Arab. J. Sci. Eng..

[12] Chandraker S., Dutt J.K., Agrawal A., Roy H., Chandrakar K., Mishra V. (2021). Development and Characterization of Epoxy-Based Polymeric Composite with Bio-particulates as Filler Material. Arabian J. Sci. Eng..

[13] Rojas-Lema S., Arevalo J., Gomez-Caturla J., Garcia-Garcia D., Torres-Giner S. (2021). Peroxide-Induced Synthesis of Maleic Anhydride-Grafted Poly (butylene succinate) and Its Compatibilizing Effect on Poly(butylene succinate)/Pistachio Shell Flour Composites. Molecules.

[14] Kuram E. (2020). UV and thermal weathering of green composites: Comparing the effect of different agricultural waste as fillers. J. Compos. Mater..

[15] El Mechtali F.Z., Essabir H., Nekhlaoui S., Bensalah M.O., Jawaid M., Bouhfid R., Qaiss A. (2015). Mechanical and thermal properties of polypropylene reinforced with almond shells particles: Impact of chemical treatments. J. Bionic Eng..

[16] Ruggiero A., Valášek P., Müller M., D’Amato R. (2019). Tribological investigation of epoxy/seed particle composite obtained from residues of processing *Jatropha Curcas* L. fruits. Compos. Part B Eng..

[17] Demiral İ., Şamdan C.A. (2016). Preparation and characterisation of activated carbon from pumpkin seed shell using H3PO4. Anadolu Univ. J. Sci. Technol. A-Appl. Sci. Eng..

[18] Aziz S.H., Ansell M.P. (2004). The effect of alkalization and fibre alignment on the mechanical and thermal properties of kenaf and hemp bast fibre composites: Part 1–polyester resin matrix. Compos. Sci Technol..

[19] Aziz S.H., Ansell M.P. (2004). The effect of alkalization and fibre alignment on the mechanical and thermal properties of kenaf and hemp bast fibre composites: Part 2—Cashew nut shell liquid matrix. Compos. Sci. Technol..

[20] Huko D., Kamau D.N., Ogola W.O. (2015). Effects of varying particle size on mechanical and combustion characteristics of mango seed shell cashew nut shell composite briquettes. Int. J. Eng. Sci. Invent..

[21] Mittal V., Sinha S. (2017). Effect of chemical treatment on thermal properties of bagasse fiber-reinforced epoxy composite. Sci. Eng. Compos. Mater..

[22] Chang W.P., Kim K.J., Gupta R.K. (2009). Moisture absorption behavior of wood/plastic composites made with ultrasound-assisted alkali-treated wood particulates. Compos. Interfaces.

[23] Fioresi F., Vieillard J., Bargougui R., Bouazizi N., Fotsing P.N., Woumfo E.D., Brun N., Mofaddel N., Le Derf F. (2017). Chemical modification of the cocoa shell surface using diazonium salts. J. Colloid. Interface Sci..

[24] John M.J., Anandjiwala R.D. (2008). Recent developments in chemical modification and characterization of natural fiber-reinforced composites. Polym. Compos..

[25] Aprilia N.S., Khalil H.A., Bhat A.H., Dungani R., Hossain M.S. (2014). Exploring material properties of vinyl ester biocomposites filled carbonized jatropha seed shell. BioResources.

[26] Garcia A., Lopez Y., Karimi K., Benitez A., Lundin M., Taherzadeh M.J., Martin C. (2015). Chemical and physical characterization and acid hydrolysis of a mixture of Jatropha curcas shells and husks. Cellul. Chem. Technol..

[27] Kabir M.M., Wang H., Lau K.T., Cardona F. (2012). Chemical treatments on plant-based natural fibre reinforced polymer composites: An overview. Compos. Part B Eng..

[28] Bazan P., Salasińska K., Kuciel S. (2021). Flame retardant polypropylene reinforced with natural additives. Ind. Crops Prod..

[29] Açıkalın K. (2012). Pyrolytic characteristics and kinetics of pistachio shell by thermogravimetric analysis. J. Therm. Anal. Calorim..

[30] Gupta S., Gupta G.K., Mondal M.K. (2020). Thermal degradation characteristics, kinetics, thermodynamic, and reaction mechanism analysis of pistachio shell pyrolysis for its bioenergy potential. Biomass Convers. Biorefinery.

[31] Açıkalın K. (2011). Thermogravimetric analysis of walnut shell as pyrolysis feedstock. J. Therm. Anal. Calorim..

[32] Mukherjee A., Ganguly P.K., Sur D. (1993). Structural mechanics of jute: The effects of hemicellulose or lignin removal. J. Text. Inst..

[33] Nayak S.K., Mohanty S., Samal S.K. (2009). Influence of short bamboo/glass fiber on the thermal, dynamic mechanical and rheological properties of polypropylene hybrid composites. Mater. Sci. Eng. A.

[34] Nuñez A.J., Kenny J.M., Reboredo M.M., Aranguren M.I., Marcovich N.E. (2002). Thermal and dynamic mechanical characterization of polypropylene-woodflour composites. Polym. Eng. Sci..

[35] Ardanuy M., Antunes M., Velasco J.I. (2012). Vegetable fibres from agricultural residues as thermo-mechanical reinforcement in recycled polypropylene-based green foams. Waste Manag..

[36] Melo P.M.A., Macêdo O.B., Barbosa G.P., Santos A.S.F., Silva L.B. (2021). Reuse of Natural Waste to Improve the Thermal Stability, Stiffness, and Toughness of Postconsumer Polypropylene Composites. J. Polym. Environ..

[37] Quillin D.T., Caulfield D.F., Koutsky J.A. (1993). Crystallinity in the polypropylene/cellulose system. I. Nucleation and crystalline morphology. J. Appl. Polym. Sci..

[38] Fatou J.G. (1971). Melting temperature and enthalpy of isotactic polypropylene. Eur. Polym. J..

[39] Bendahou A., Kaddami H., Sautereau H., Raihane M., Erchiqui F., Dufresne A. (2008). Short palm tree fibers polyolefin composites: Effect of filler content and coupling agent on physical properties. Macromol. Mater. Eng..

[40] Levytskyi V.E., Masyuk A.S., Bilyi L.M., Bialopiotrowicz T., Humenetskyi T.V., Shybanova A.M. (2020). Influence of Silicate Nucleation Agent Modified with Polyvinylpyrrolidone on the Morphology and Properties of Polypropylene. Mater. Sci..

[41] Sreenivasan S., Iyer P.B., Iyer K.K. (1996). Influence of delignification and alkali treatment on the fine structure of coir fibres (*Cocos nucifera*). J. Mater. Sci..

[42] Lin N., Fan D., Chang P.R., Yu J., Cheng X., Huang J. (2011). Structure and properties of poly (butylene succinate) filled with lignin: A case of lignosulfonate. J. Appl. Polym. Sci..

